# Semantic interrogation of a multi knowledge domain ontological model of tendinopathy identifies four strong candidate risk genes

**DOI:** 10.1038/srep19820

**Published:** 2016-01-25

**Authors:** Colleen J. Saunders, Mahjoubeh Jalali Sefid Dashti, Junaid Gamieldien

**Affiliations:** 1 South African National Bioinformatics Institute/MRC Unit for Bioinformatics Capacity Development, University of the Western Cape, Bellville, South Africa

## Abstract

Tendinopathy is a multifactorial syndrome characterised by tendon pain and thickening, and impaired performance during activity. Candidate gene association studies have identified genetic factors that contribute to intrinsic risk of developing tendinopathy upon exposure to extrinsic factors. Bioinformatics approaches that data-mine existing knowledge for biological relationships may assist with the identification of candidate genes. The aim of this study was to data-mine functional annotation of human genes and identify candidate genes by ontology-seeded queries capturing the features of tendinopathy. Our BioOntological Relationship Graph database (BORG) integrates multiple sources of genomic and biomedical knowledge into an on-disk semantic network where human genes and their orthologs in mouse and rat are central concepts mapped to ontology terms. The BORG was used to screen all human genes for potential links to tendinopathy. Following further prioritisation, four strong candidate genes (*COL11A2*, *ELN*, *ITGB3*, *LOX*) were identified. These genes are differentially expressed in tendinopathy, functionally linked to features of tendinopathy and previously implicated in other connective tissue diseases. In conclusion, cross-domain semantic integration of multiple sources of biomedical knowledge, and interrogation of phenotypes and gene functions associated with disease, may significantly increase the probability of identifying strong and unobvious candidate genes in genetic association studies.

Tendons and ligaments are connective tissue structures that are frequently injured during participation in physical activity and repetitive activities in the workplace^1^,^2^. Tendinopathy is defined as a multifactorial clinical syndrome marked by tendon pain, thickening and impaired performance^3^,^4^,^5^. Whilst the aetiology of tendinopathy is still poorly understood, it is clear that the condition is complex and multifactorial in nature^2^,^6^. As with many other orthopaedic conditions, a number of intrinsic risk factors contribute to an individual’s predisposition to developing tendinopathy. Extrinsic risk factors further increase a predisposed individuals susceptibility to developing tendinopathy when exposed to an inciting stimulus^7^,^8^. Genetic risk factors contribute to intrinsic predisposition through direct effects on tendon tissue, as well as indirectly through heritable variation in other intrinsic risk factors such as flexibility, sex, adiposity and anthropometry^7^. The genetic contribution to tendinopathy predisposition is therefore polygenic in nature and the identification of each new genetic risk factor improves the ability of risk models to discriminate risk of tendinopathy^9^.

The current cost of genome wide association studies and next generation methodologies such as whole exome and whole genome sequencing deter their use in investigating genetic risk factors for many lower priority diseases and conditions. To date, the predominant approach in tendinopathy has been that of hypothesis driven case-control genetic association studies in which a candidate gene and candidate variants have been identified for investigation. In recent years, several tendinopathy candidate genes have been investigated resulting in the successful identification of genetic variants that contribute to risk with moderate to large effect sizes^2^,^7^. Several other studies have found no association between the candidate variants investigated and the condition^10^,^11^,^12^,^13^. However, it is worth noting that using this approach, the lack of association with a candidate variant does not exclude other variants within that gene as contributors to tendinopathy risk. Although candidate genes (and variants) are selected for investigation based on biological function and *a priori* hypotheses that their protein products are involved in the aetiology of the studied condition, it is possible that many strong candidate genes may be missed using this approach. In particular, genes encoding proteins with transitive links to tendon biology and pathophysiology may not be immediately obvious as good candidate genes. Bioinformatics approaches that data-mine existing knowledge, annotated and stored within public databases, for interactions and biological relationships would serve to significantly increase the probability of identifying such genes.

We have developed the BioOntological Relationship Graph (BORG) system^14^, which integrates multiple sources of genomic and biomedical knowledge into an on-disk semantic network where human genes and their orthologs in mouse and rat are central concepts. By modelling relationships between phenotypes and functions associated with disease, it uses a ‘guilt-by indirect-association’ semantic discovery engine to find unobvious yet biologically plausible and literature-supported transitive gene-to-disease associations through *in silico* experimentation. We hypothesize that integration of such bioinformatics tools with traditional molecular biology approaches may assist in identifying the potential sources of missing heritability in tendinopathy. The aim of this study was, therefore, to develop a semantic model of tendinopathy in the BORG database in order to data-mine functional annotation of human genes and identify potential candidate genes by ontology-seeded queries that capture the known phenotypes and features of tendinopathy.

## Results

Of the known human genes (n = 20661) passed through the tendinopathy specific BORG, 608 were identified as having annotated links to one or more ontology terms describing features of tendinopathy and were considered as preliminary candidate genes (Supplementary Table 1). The subset of 277 candidates that had at least two independent ontology links to ‘tendinitis’ were enriched for involvement in connective tissue (*p* = 7.5E^−36^), musculoskeletal (*p* = 2.2E^−26^) and vascular diseases (*p* = 4.07E^−23^). Of these, eight have previously been implicated in all three disease types (Fig. 3)^15^. Further prioritisation of these candidate genes revealed that (i) 137 have previously been implicated in other connective tissue diseases, (ii) 251 exhibited more than ten independent ontology links to tendinopathy and, (iii) 84 were linked to tendinopathy through multiple knowledge domains. Whilst none of these prioritisation strategies exclude any genes from being candidates, 35 were found to meet all three criteria and were considered to be strong candidate genes (Table 2). The STRING database^16^ showed that the protein products of 28 of these strong candidate genes were involved in a physical or functional interaction with at least one other in the set, of which 24 were predicted to form a distinct interaction network (Fig. 4).

Reanalysis of the GDS4901 dataset^17^ deposited in NCBI’s Gene Expression Omnibus yielded 92 genes that were differentially expressed in diseased tendons compared to their healthy control tendons after correcting for multiple testing (Supplementary Table 2). Further prioritisation of these differentially expressed genes revealed that (i) five have previously been implicated in other connective tissue diseases, (ii) five exhibited more than ten independent ontology links to features or functions associated with tendinopathy and, (iii) five were linked to tendinopathy through multiple knowledge domains. Four of these genes (*COL11A2; ELN; ITGB3; LOX*) were found to meet all three prioritisation criteria (Table 2).

## Discussion

The current study identified four strong candidate genes (*COL11A2; ELN; ITGB3; LOX*) that are differentially expressed in tendinopathy, functionally linked to features of tendinopathy and previously implicated in the aetiology of other connective tissue diseases. The *LOX* gene encodes lysyl oxidase (LOX), a copper-dependant enzyme which facilitates covalent cross-linking of both collagen and elastin fibres in connective tissue. Recent evidence suggests that the density of LOX mediated cross-linking is correlated with mechanical properties of embryonic tendon tissue^18^, and the inhibition of LOX activity results in irregular collagen fibrils of wide ranging fibril diameter without affecting the total collagen content of tissue^19^. In essence, this enzyme plays a non-redundant role in the early establishment of collagen network integrity in connective tissues^19^. Interestingly, the gene encoding a *LOX* homolog, lysyl oxidase like-1 (*LOXL1*), was also identified as a candidate gene in this study. LOXL1 is specifically localised to sites of elastogenesis and non-redundantly facilitates the cross-linking of tropoelastin into elastin fibres^20^. In these elastic fibres LOXL1 interacts with a scaffold of fibulin-5 (encoded by the *FBLN5* gene) to ensure spatially delineated elastin deposition in the extracellular matrix^20^. In the GDS4901 expression dataset analysed in this study, the *LOX* (1,97 FC; p < 0,001), *LOXL1* (2,34 FC; p < 0,0001) and *ELN* (1,84 FC; p < 0,0001) genes were all up-regulated in tendinopathy (Supplementary Table 2), and *FBLN5* is linked to tendinopathy through its role in extracellular matrix organisation in humans as well as in rat and mouse knockout models (Supplementary Table 1).

*ELN*, the gene encoding the soluble protein elastin, was also identified as a strong candidate gene in this study. Elastin contributes approximately 2% of the dry weight of tendon and is one of the primary structural components of elastic fibres in connective tissue. El Khoury *et al*. (2015)^13^ previously investigated the rs2071307 candidate variant within the *ELN* gene and found it not to be associated with Achilles tendinopathy. However, the transcriptomic findings and BORG semantic annotations presented here, as well as the fact that it is involved in three disease classes that are related to tendinopathy (Fig. 4), strongly suggests that other functional or regulatory variants in this gene may contribute to risk.

A third strong candidate gene, *ITGB3*, encodes glycoprotein IIIa (GP IIIA), a subunit of several integrins and receptor complexes within the extracellular matrix. In particular, GP IIIA forms a subunit of integrin α_v_β_3_ which is an important regulator of pathological angiogenesis^21^. Integrin α_v_β_3_ specifically regulates vascular endothelial growth factor receptor-2 (VEGFR2) function and therefore has wide ranging downstream effects in cellular functions involving vascular endothelial growth factor^21^. VEGFR2, also known as the kinase insert domain receptor, is encoded by the *KDR* gene which has itself been implicated in anterior cruciate ligament ruptures^22^. The cellular integrin α_v_β_3_ - growth factor interface is key in a number of outside-in signalling pathways^21^ making integrin α_v_β_3_ a good upstream candidate gene in the angiogenesis-associated signalling pathways. The *ITGB3* gene also occupies a central position in the functional interaction network (Fig. 4) predicted from the strong candidate genes in Table 2 by the STRING database. This further highlights its’ likely importance in tendon biology.

The gene encoding the alpha-2 strand of type XI collagen (*COL11A2*) was also identified as a strong candidate gene in this study. This gene is significantly down-regulated in tendinopathy samples (−1,85 FC; p < 0,0005) and is functionally linked to the condition through its role in collagen fibril and extracellular matrix organisation. Type XI collagen is particularly important in developing tendon where it interacts with type V collagen to regulate early fibrillogenesis^23^. In addition, it is strongly associated with a number of other connective tissue diseases such as Stickler syndrome^24^. Hay *et al*. have previously investigated a *COL11A2* variant, rs1799907, in tendinopathy and found it to be included in a pseudohaplotype, but not independently associated with Achilles tendinopathy^25^.

A number of other strong candidate genes were also identified that are implicated in connective tissue diseases and have numerous annotated links to the condition, but are not differentially expressed in tendinopathy samples (Table 2). These include genes encoding collagen alpha-chains (*COL1A1*, *COL1A2*, *COL2A1*, *COL3A1*, *COL5A1, COL5A2* and *COL11A1*) and members of the transforming growth factor family (*BMP2*, *TGFB2*), as well as genes involved in glycoprotein and proteoglycan metabolism (*ACAN*, *B4GALT1, COMP, FOXC1, HPSE, HSPG2*), collagen metabolism (*ADAMTS2*, *MMP9, SERPINH1, TNXB)*, angiogenesis (*ANGPT2, CYR61, FGF2, HIF1A, IL1B, ITGB2, PTK2B*) and inflammation (*CX3CL1, CXCR3*). Although expected, it is worthwhile noting that the semantic discovery strategy was able to identify as candidate genes several genes that have previously been associated with tendinopathy (*ADAMTS14*, *COL5A1*, *COL11A1*, *FBN2*, *TIMP2*, *TNC*)^11^,^13^,^25^,^26^,^27^,^28^,^29^  and anterior cruciate ligament ruptures (*ACAN*, *BGN*, *DCN*, *KDR*)^22^,^30^. However, not all of these genes were identified as strong candidate genes in subsequent prioritisation steps. For example, a number of variants within the *TNC* gene encoding Tenascin-C are associated with Achilles tendinopathy^26^,^28^, however *TNC* was not prioritised as a strong candidate gene in this study. Another interesting example is the *SCX* gene, which encodes the transcription factor scleraxis. Recent evidence suggests that scleraxis is a marker of tendon progenitor cells and plays a vital role during development of these tissues^3^,^31^. The short list of strong candidate genes (Table 2) therefore highlights genes meeting all three prioritisation criteria but should not be viewed as exhaustive. In particular, candidate genes that have not previously been associated with other connective tissue diseases may still be strong candidates, and may in fact have Achilles tendinopathy-specific roles. It should additionally be noted that several genes previously associated with Achilles tendinopathy (*CASP8*; *IL-6*; *IL-1RN*)^12^,^32^ were not identified as candidate genes at all. This could indicate missing gene annotations in the databases or, more likely, an incompleteness of the semantic model for tendinopathy used to capture biological pathways, functions and knock out phenotypes likely to be relevant to the aetiology. Future versions of this model should consider the inclusion of ontology terms capturing specific inflammatory signalling pathways^12^,^32^,^33^. There is, however, a trade-off between the sensitivity of the model to capture all candidate genes and the specificity of the model. Including more ontology terms, or broader terms, in the model, can increase sensitivity but will also decrease the specificity of the model. For example, the *SERPINE1* gene (encoding an endothelial plasminogen activator inhibitor) was identified in this study as a strong candidate gene based on the number of independent and unique annotated paths linking it to angiogenesis and, therefore, ‘tendinitis’. Subjective inspection of gene function shows that, although involved in angiogenesis, the protein product functions more specifically in controlling fibrinolysis^34^, a process not likely to be involved in the aetiology of tendinopathy. However, a sensitive model with less stringent prioritisation criteria allows for the identification of unobvious candidate genes with transitive links to tendinopathy. In particular, by prioritising candidate genes using only the number of independent and unique semantic pathways identified, several such genes were highlighted. For example, the *ATP7A* gene is proposed by the system to be linked to tendinopathy through four different ontology paths. This gene encodes a transmembrane copper-transporting enzyme that may play an important role in the functioning of the extracellular copper enzyme, lysyl oxidase^35^, one of the four strongest candidates identified in this study.

In conclusion, four strong candidate genes (*COL11A2; ELN; ITGB3; LOX)* were identified as differentially expressed in tendinopathy, functionally linked to features of tendinopathy and implicated in the aetiology of other connective tissue diseases. Our findings strongly suggest that cross-domain semantic integration of multiple sources of biomedical knowledge and molecular data and the modelling of phenotypes and gene functions associated with disease, may significantly increase the probability of identifying strong and unobvious candidate genes in both hypothesis driven and hypothesis generating genetic association and omics studies. The investigation of both the molecular and genomic interactions of these candidate genes in tendinopathy is a promising area of further research.

## Methods

### BioOntological Relationship Graph (BORG) database

Our BioOntological Relationship Graph (BORG) database leverages knowledge representation theories and the superior ‘real-world’ modelling capabilities of the Neo4J graph database management system to integrate disparate biomolecular and biomedical facts, observations and extant knowledge from human, rat and mouse into a single large on-disk semantic network. The system assimilates and integrates multiple sources of genomic and biomedical knowledge and metadata and is able to learn rules about diseases including the phenotypes and gene functions associated with disease. It has a custom query facility that uses individual units of knowledge and the links between them to answer complex questions that require the simultaneous interrogation of multiple knowledge domains. The functionality used in this study enables the uncovering of non-obvious, often transitive, yet biologically plausible and literature-supported associations between genes and diseases.

### Building the BORG core

**Figure 1:**
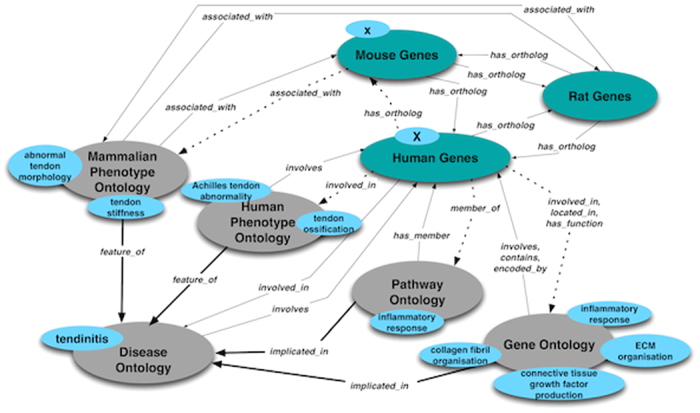
Tendinopathy specific BioOntological Relationship Graph (BORG) semantic schema. Heavy solid arrows indicate the mapping of phenotypes and functions known to be features of tendinopathy with the disease ontology term “tendinitis”. Dashed arrows indicate the possible direct and transitive paths by which a hypothetical human gene “*X*” may be linked to “tendinitis”.

The BORG database is structured as a schema-free semantic network, which acts as a model of associative memory representing biological and biomedical concepts and the natural relationships between them to represent relevant existing knowledge. Through the use of the Neo4j graph database management system (neo4j.org), information is directly stored as a directed acyclic graph (DAG), where nodes represent concepts that can have any number of attributes and the edges in the graph are directed and represent relationships between concepts, which can also have any number of attributes. In most cases, we represent a relationship between two concepts by a pair of opposing directed edges between them in order to explicitly represent a fact or a single unit of knowledge. For example, gene *X* is “involved_in” biological process Y and biological process Y “involves” gene *X*. The advantage of our schema free approach is that entire new knowledge domains can instantly be assimilated by a graph database, as long as a strong semantic model is adhered to. While any concept/node in the graph database can be associated with any other, we ensured that the knowledge and facts in the database were represented in a semantically correct manner coinciding with the way a scientist would understand them. A semantic model (Fig. 1) of the real world relationships between the concept classes was developed to enforce this, but it is important to reiterate that the model is schema-free.

At the conceptual centre of the semantic network are human, mouse and rat genes, each having an orthology edge between it and its counterpart in the other species where applicable. It is important to note that in the use case represented by this study, each gene ‘inherits’ knowledge linked to its counterpart in the other species’. A human gene would, for example, be transitively associated with a phenotype that arises in a mouse or rat gene knockout model of the orthologous gene. At the periphery of the semantic network database graph are formal biological ontologies from the OBO Foundry (http://www.obofoundry.org/):

1. The Gene Ontology (GO)^36^ describes the function of gene products in terms of their cellular location, their molecular functions and the biological processes they are involved in. GO annotations for genes from the three species were downloaded from NCBI (http://ftp.ncbi.nlm.nih.gov/gene/DATA/gene2go.gz).
2. The Pathway Ontology^37^ describes all types of canonical and altered pathways at a semantic rather than structural level, as well as the relationships between them. Gene to pathway links were downloaded from the Rat Genome Database FTP site (ftp://rgd.mcw.edu/pub/data_release/).
3. The Disease Ontology^38^ is a comprehensive hierarchical controlled vocabulary aimed at enabling interoperability between biological and clinical human disease descriptors. Gene to disease associations were obtained from the project web site.
4. The Human Phenotype Ontology^39^ is a very widely used structured controlled vocabulary for the phenotypic features encountered in human diseases and is aimed at linking molecular biology and disease through phenotype data. Gene to phenotype links were obtained from the project web site.
5. The Mammalian Phenotype Ontology^40^ enables robust annotation of mammalian phenotypes in the context of genetic variations and gene knockout models that are used as models of human biology and disease. Mouse and rat gene to phenotype links were downloaded from the Rat Genome Database FTP site (ftp://rgd.mcw.edu/pub/data_release).

In practice, the semantic database is loaded in a stepwise manner to ensure consistency and traceability. First, genes and all their relevant attributes were loaded. Orthology edges were then inserted between each gene and its counterpart in the other two species. Ontologies were loaded in a similar manner, one at a time, where terms and their attributes were inserted first, followed by annotated edges describing the relationships defined in the OBO file. Lastly, genes were mapped to appropriate ontology terms based on the gene-to-term relationships provided by the various ontology projects described above. This represented the core database, in which disease-specific models could be represented based on known or hypothesised relationships between a disease term and terms in other ontologies that describe various features of the disease.

### Semantic modelling of tendinopathy in the BORG database

In order to model specific diseases we used a concept of cross-ontology linking, where terms from ontologies representing separate knowledge domains are related via an edge. Specifically, additional edges were added to the core database to represent relationships of gene functions, phenotypes and pathways to human diseases (Fig. 1), resulting in a richer semantic network in which genes are transitively associated with a disease of interest. In this study, ontology terms capturing the known features of tendinopathy^4^,^6^,^41^ were identified by keyword searching the BORG database, as well as the Ontology Lookup Service^42^. These ontology terms (Table 1) were semantically linked to, and annotated as “feature_of”, the most appropriate DOID term for tendinopathy. The DOID term “tendinitis” (DOID:971) was chosen as that term which most closely describes tendinopathy. While the authors acknowledge the debate surrounding the use of the term “tendinitis”^4^,^33^,^43^, it should be noted that this DOID term is used only in the context of seeding database queries and that the ontology terms and phenotypes semantically linked to this DOID term are those that capture the consensus features of human tendinopathy^4^,^6^,^41^. An important advantage of using standard ontologies is that concepts associated to identified ontology terms (Table 1) via their child terms will be identified through a transitive closure on the underlying graph data structure. For example, genes previously associated with ‘tenosynovitis’, ‘tibialis tendinitis’ and ‘patellar tendinitis’ will all be correctly associated with the term of interest, ‘tendinitis’. Similarly, genes associated with ‘regulation of fibril organization’ and ‘amyloid fibril formation’ would automatically be linked to the disease of interest via their grandparent term ‘ECM organisation’ which was used in constructing the semantic model.

### Graph path-based semantic discovery

**Figure 2:**
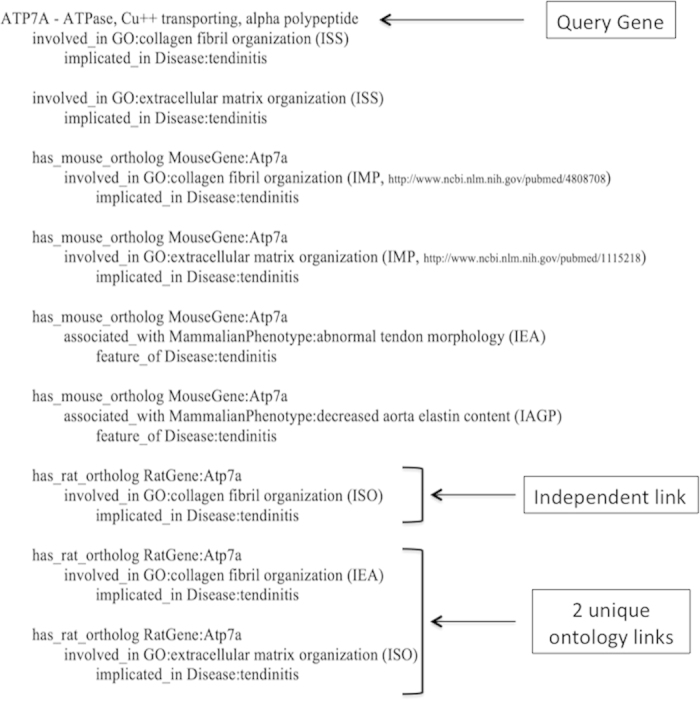
Sample output for the human gene, *ATP7A*. BioOntological Relationship Graph (BORG) output for each human gene passed to it lists each direct and/or transitive path between that gene and the disease of interest (e.g. “tendinitis”). Evidence codes: IAGP = Inferred by association of genotype with phenotype, IEA = Inferred from electronic annotation, IMP = Inferred from mutant phenotype, ISO = Inferred from sequence orthology, ISS = Inferred from sequence or structural similarity.

**Figure 3 f3:**
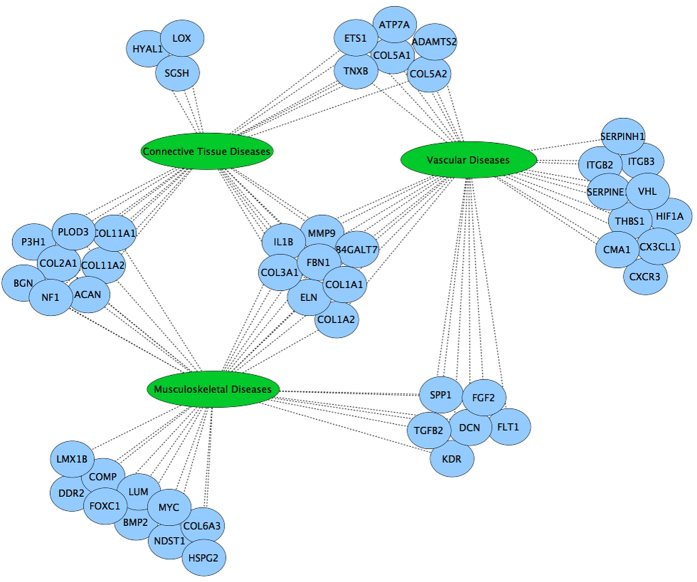
Involvement of candidate genes in related disease classes. Genes having at least two ontology links to tendinitis were further annotated based on their known roles in connective tissue, musculoskeletal and vascular diseases.

**Figure 4 f4:**
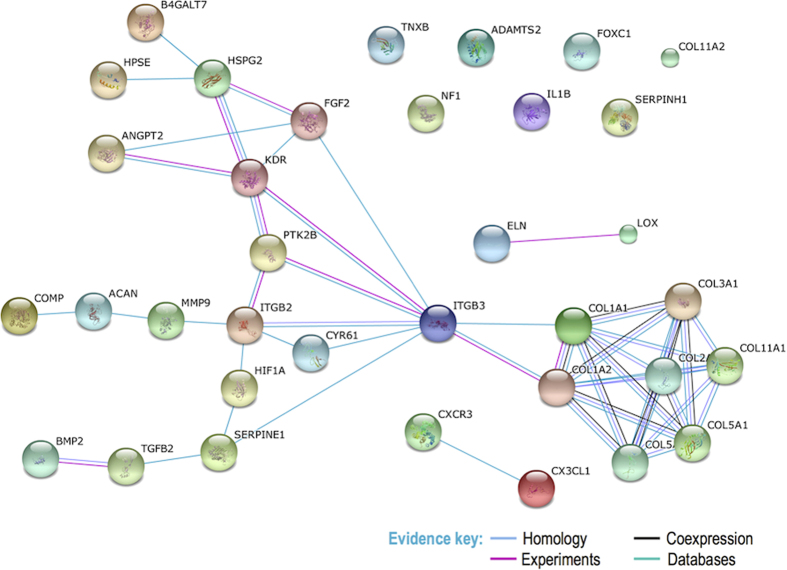
STRINGdb generated interaction network of top candidates.

The BORG query used in this study provides the ability to find transitive links between source and target concepts, while simultaneously explaining the biological relevance of the link in a natural language result (Fig. 2). When used for finding potential links between genes and disease, the BORG performs a directed walk on the graph to find all allowed paths between a gene of[Fig f3]interest and a specified disease term. There are three possible path[Fig f4]queries: (i) report the shortest path(s), (ii) report all paths of a pre-specified length and (iii) report all paths of any length between a gene of interest and the disease term of interest. Where available, all queries return evidence codes as well as links to the relevant scientific publication from which the stored fact was obtained. By restricting the edges which may be followed, this query on the semantic network is able to identify gene-to-disease links that are non-obvious yet still make biological sense. To simplify, the graph walk is only allowed in one direction: away from the gene towards the disease term of interest, either directly or via terms and edges that are deemed relevant to the disease.

The benefit of a disease specific semantic model is that it enables identification of new genes that are functionally similar to known disease genes, and the ability to score proposed associations based on the number of links and the number of knowledge domains contributing to the association. Reports are produced on a per-gene basis and are particularly useful when filtering a large list of candidates, since only genes that have at least one path leading to the disease will be returned. The report itself is self-explanatory and provides the researcher with substantial amounts of information from multiple knowledge domains which can be used to manually prioritize the remaining candidates. The formal rigorousness of the biological ontologies and the ‘guilt-by-indirect-association’ approach thus ensures that genes that may otherwise have been overlooked as candidates when directly consulting the literature or individual databases are automatically selected in a biologically and semantically relevant manner. This is also the main differentiator between BORG and Phenolyzer^44^, the tool that, to our knowledge, is closest in functionality to BORG. Figure 2 shows an example output when querying the database for annotated paths linking the *ATP7A* gene to the disease term “tendinitis”.

### Identification of candidate tendinopathy genes

The database of all known human genes was downloaded from the NCBI database (ftp://ftp.ncbi.nlm.nih.gov/gene/DATA/GENE_INFO/Mammalia/Homo_sapiens.gene_info.gz; January 2015) and the corresponding list of approved HUGO Gene Nomenclature Committee (HGNC) gene symbols was extracted. This list of genes was passed to the tendinopathy specific BORG database to extract the reduced set of human genes with direct or transitive links to the ontology terms designated as features of tendinopathy (Figure 1). This gene set formed the basic set of candidate genes for investigation in tendinopathy. In order to assess the quality of candidates identified by our semantic discovery approach, genes having at least two ontology links to tendinitis were subjected to disease enrichment analysis by the Comparative Toxicogenomics Database’s webservice^15^ using a Bonferroni adjusted p-value of 0.01 as the statistical significance threshold. Subsequently, a network infographic depicting overlapping involvement of these candidate genes in connective tissue (MESH id: D003240), musculoskeletal (MESH id: D009140) and vascular diseases (MESH id: D014652) was produced based on these results.

Intuitive BORG queries were used to prioritise candidate genes in a biologically relevant manner. These included prioritising genes with more than ten independent annotated paths to ‘tendinitis’ as a measure of certainty, and/or genes linked to ‘tendinitis’ by multiple knowledge domains. In addition, genes previously implicated in other connective tissue diseases (DOID:65) were also prioritised. The DOID term “connective tissue disease” is defined as “A musculoskeletal system disease that affects tissues such as skin, tendons, and cartilage” and includes, but is not limited to, collagen diseases, bone diseases, enthesopathies, fasciitis and bursitis^42^. STRINGdb v10^16^ was used to generate a functional interaction network of these top candidates, using only high confidence interactions (STRING score > = 0.7) for network construction.

### Differential gene expression in tendinopathy

In addition to functional variants within coding regions of genes linked to tendinopathy, functional variants within regulatory regions of differentially expressed genes may also be implicated as genetic risk factors for tendinopathy. A public gene expression dataset was therefore used to investigate differential gene expression in tendinopathy. The GDS4901 dataset, submitted by Jelinsky *et al*. (2011)^17^ was downloaded from NCBI’s Gene Expression Omnibus. This dataset contains gene expression data from the Affymetrix Human Genome U133 Plus 2.0 Array platform for 23 pairs of diseased and healthy tendons from patients undergoing surgical treatment for chronic tendinopathy. Macro- and microscopic analysis confirmed histological characteristics of tendinopathy in all tendinopathy samples^17^. Microarray analysis was performed using the ‘affy’ and ‘limma’ Bioconductor packages in the R programming environment^45^,^46^. The Benjamini and Hochberg correction for multiple testing was used to adjust p-values^47^. Genes were considered differentially expressed if average fold change in tendinopathy was >1.5 or <−1.5, and the adjusted p-value was <0.05. The list of differentially expressed genes was passed to the tendinopathy-specific BORG database to extract the set of genes with direct or transitive links to ‘tendinitis’ (Figure 1). Candidates from this analysis were further prioritised by screening for genes that had more than ten independent annotated paths to ‘tendinitis’, genes linked to ‘tendinitis’ by multiple knowledge domains, and genes previously associated with other connective tissue diseases.

## Additional Information

**How to cite this article**: Saunders, C. J. *et al.* Semantic interrogation of a multi knowledge domain ontological model of tendinopathy identifies four strong candidate risk genes. *Sci. Rep.* **6**, 19820; doi: 10.1038/srep19820 (2016).

## Supplementary Material

Supplementary Table 1

Supplementary Table 2

## Figures and Tables

**Table 1:** Ontology terms linked to Tendinitis (DOID:971) to create a cross-ontology semantic model of tendinopathy in the BioOntological Relationship Graph (BORG) database.

TERM ID	ONTOLOGY TERM
*Gene Ontology*	
GO:0035989	Tendon development
GO:0030199	Collagen fibril organisation
GO:0045766	Positive regulation of angiogenesis
GO:0060055	Angiogenesis involved in wound healing
GO:0044346	Fibroblast apoptotic process
GO:0030203	Glycosaminoglycan metabolic process
GO:0006029	Proteoglycan metabolic process
GO:0032963	Collagen metabolic process
GO:0009100	Glycoprotein metabolic process
GO:0030198	ECM organisation
*Mammalian Phenotype Ontology*	
MP:0003198	Calcified tendon
MP:0011643	Abnormal tendon collagen fibril morphology
MP:0005503	Tendon dysplasia
MP:0003907	Abnormal tendon stiffness
MP:0005601	Increased angiogenesis
MP:0003710	Abnormal physiological neovascularization
MP:0011475	Abnormal glycosaminoglycan level
*Human Phenotype Ontology*	
HP:0004690	Thickened Achilles tendon
HP:0005197	Generalised morning stiffness
HP:0011988	Ectopic ossification in tendon tissue

**Table 2:** The 35 strongest candidate genes for association with tendinopathy identified by cross knowledge domain semantic discovery in the BioOntological Relationship Graph (BORG) database.

HGNC Gene Symbol	Ensembl Gene ID	No. of independent paths to “Tendinitis^#^”	No. of unique paths to “Tendinitis^#^”	Fold change expression in tendinopathic vs healthy tendons^*^
*ACAN*	ENSG00000157766	22	3	
*ADAMTS2*	ENSG00000087116	22	2	
*ANGPT2*	ENSG00000091879	16	2	
*B4GALT7*	ENSG00000027847	22	2	
*BMP2*	ENSG00000125845	22	2	
*COL11A1*	ENSG00000060718	76	4	
*COL11A2*	ENSG00000204248	28	2	−1.85
*COL1A1*	ENSG00000108821	22	2	
*COL1A2*	ENSG00000164692	22	2	
*COL2A1*	ENSG00000139219	48	3	
*COL3A1*	ENSG00000168542	30	2	
*COL5A1*	ENSG00000130635	62	3	
*COL5A2*	ENSG00000204262	30	2	
*COMP*	ENSG00000105664	23	2	
*CX3CL1*	ENSG00000006210	40	2	
*CXCR3*	ENSG00000186810	23	2	
*CYR61*	ENSG00000142871	26	2	
*ELN*	ENSG00000049540	20	2	1,84
*FGF2*	ENSG00000138685	34	2	
*FOXC1*	ENSG00000054598	40	2	
*HIF1A*	ENSG00000100644	22	2	
*HPSE*	ENSG00000173083	19	4	
*HSPG2*	ENSG00000142798	16	2	
*IL1B*	ENSG00000125538	26	2	
*ITGB2*	ENSG00000160255	14	2	
*ITGB3*	ENSG00000259207	28	5	1,68
*KDR*	ENSG00000128052	37	3	
*LOX*	ENSG00000113083	37	3	1,97
*MMP9*	ENSG00000100985	44	4	
*NF1*	ENSG00000196712	46	2	
*PTK2B*	ENSG00000120899	15	2	
*SERPINE1*	ENSG00000106366	31	3	
*SERPINH1*	ENSG00000149257	22	2	
*TGFB2*	ENSG00000092969	40	2	
*TNXB*	ENSG00000168477	40	3	

Genes listed were previously implicated in other connective tissue diseases and subsequently subjected to further prioritisation strategies. *GEO dataset GDS4901; p < 0,05 ^#^Human disease ontology term (DOID:971)
